# Comparison of Mass Spectrometry Imaging by Desorption Electrospray Ionization (DESI) and Desorption Electro-Flow Focusing Ionization (DEFFI)

**DOI:** 10.3390/metabo16040219

**Published:** 2026-03-27

**Authors:** Yunshuo Tian, Ruolun Wei, Yifan Meng, Richard N. Zare

**Affiliations:** 1 College of Letter and Science, University of California, Santa Barbara, CA 93106, USA; 2 Department of Neurosurgery, Stanford University School of Medicine, Stanford, CA 94305, USA; rlwei@stanford.edu; 3 Department of Chemistry, Stanford University, Stanford, CA 94305, USA

**Keywords:** mass spectrometry imaging, desorption electrospray ionization, desorption electro-flow focusing ionization, spatial resolution, imaging speed

## Abstract

Background: Among atmospheric-pressure mass spectrometry imaging (MSI) methods, desorption electrospray ionization (DESI) and desorption electro-flow focusing ionization (DEFFI) represent cost-effective, high-throughput approaches that utilize pneumatically assisted charged solvent droplets to directly desorb and ionize analytes from sample surfaces. Methods and Results: In this study, we systematically compare the performance of conventional DESI-MSI with previously reported DEFFI-MSI configurations on the Orbitrap mass spectrometer platform, focusing on evaluating the lateral spatial resolution, signal intensity, and imaging speed. By scanning a standard patterned sample which has sharp edges, DESI-MSI achieved a spatial resolution of 70 µm, while DEFFI-MSI achieved 15 µm (approximately 4.7-fold improvement). For the representative ion at *m*/*z* 782.5621, DEFFI-MSI demonstrated significantly higher signal intensity across solvent flow rates ranging from 0.5 to 1.5 µL min^−1^. The enhanced ion yield directly translates to improved Orbitrap-based MSI efficiency: in both negative- and positive-ion modes, DEFFI generates rich full-scan mass spectra within the maximum 10 ms ion injection time, whereas DESI produces weaker mass spectra under the same conditions. Conclusions: Taken together, these results quantify the key performance metrics between DESI-MSI and DEFFI-MSI, demonstrating that DEFFI is the preferred method on Orbitrap-based MSI, because it simultaneously enhances spatial resolution, signal intensity, and imaging speed.

## 1. Introduction

Ambient pressure mass spectrometry imaging (AP-MSI) enables spatially resolved chemical analysis directly from surfaces with minimal sample preparation, making it attractive for rapid profiling of biological tissues and other complex materials [1,2,3,4,5,6,7,8,9,10]. In practical AP-MSI workflows, three performance parameters dominate method selection: spatial resolution, ion yield, and imaging throughput. These parameters together determine how much signal can be generated per unit area and per unit time.

For spatial resolution, a central limitation in droplet-based MSI is that nominal pixel size is not equivalent to true spatial resolution. The true lateral resolution depends on the effective interaction region between the spray and the surface, which is governed by droplet momentum, solvent spreading, splash dynamics, and local surface properties [8]. Consequently, a method can be programmed at small step sizes while still producing blurred chemical boundaries if the sampling spot is larger than the pixel pitch. For rigorous comparisons, spatial resolution should therefore be quantified using a consistent and objective criterion, rather than inferring from pixel size alone.

Imaging throughput is another important factor, particularly in Orbitrap-based MSI. Orbitrap analyzers provide high mass-resolving power and accurate mass measurements, which are advantageous for complex biological samples and for distinguishing isobaric interferences [11]. However, Orbitrap acquisition speed is limited by the generated ion amount: when the incoming ion current is low, the instrument requires a longer ion accumulation time, which is named the ion injection time, to reach the desired ion population, increasing the time required per imaging pixel. This effect is exacerbated in ambient sources where the ion yield and ion transfer efficiency may be modest, and it can dominate the total imaging time even when stage motion could otherwise support a faster acquisition rate. In other words, for many AP-MSI experiments on Orbitrap systems, improving ion yield is not only about sensitivity, but also directly determines whether fast imaging is feasible.

Among AP-MSI techniques, desorption electrospray ionization (DESI) and desorption electro-flow focusing ionization (DEFFI) are two economical, high-utility, droplet-based approaches that rely on pneumatically assisted charged solvent droplets to desorb and ionize analytes from surfaces. DESI was originally introduced as an ambient desorption/ionization method and rapidly became widely adopted due to its simplicity and broad applicability [3,12,13,14,15,16,17]. DEFFI was later reported as an electro–flow-focusing configuration capable of generating energetic, highly charged droplets for ambient desorption/ionization [18,19,20,21]. In the imaging process, both configurations share a common conceptual workflow, spray-mediated surface extraction and ion formation. However, they differ in spray formation geometry and ion production characteristics, which may translate to measurable differences in imaging performance, including spatial resolution, ion yield, and imaging speed. A previous study suggests that DEFFI can increase ion yield and spatial resolution compared with classic DESI techniques, which should translate into shorter injection times and higher throughput on high-resolution instruments [21]. At the same time, DEFFI’s more focused spray formation can effectively reduce the sampling spot, offering a route to improve the spatial resolution without sacrificing signal intensity [20,21].

In this study, we present a systematic comparison of conventional DESI-MSI and the DEFFI-MSI configuration on an Orbitrap mass spectrometer platform using performance parameters that directly determine imaging quality and throughput. Spatial resolution is quantified on a standard patterned sample which has multiple sharp edges, providing an objective evaluation rather than pixel size. Ion yield is evaluated under imaging-relevant spray conditions by measuring representative ion signal strengths as a function of solvent flow rate. The Orbitrap acquisition speed is assessed by examining spectral quality and signal abundance across a range of maximum ion injection times in both negative- and positive-ion modes. Together, these measurements define practical performance differences between DESI-MSI and DEFFI-MSI and provide guidance for optimizing droplet-based AP-MSI when both spatial resolution and Orbitrap throughput are critical.

## 2. Materials and Methods

### 2.1. Mouse Brain Sample Preparation

A female C57BL/6 mouse was anesthetized before being decapitated. The brain tissue was dissected, embedded in CMC (carboxymethyl cellulose), and frozen at −80 °C. The mouse brain tissue sections of 7 μm thickness were obtained with a cryostat (Leica CM1900, Wetzlar, Germany). Sections were mounted on glass slides.

### 2.2. DESI and DEFFI MSI

**Figure 1:**
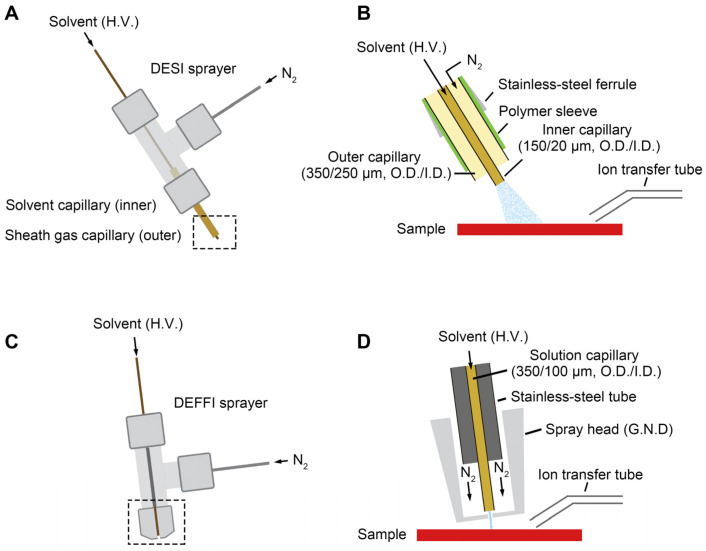
Experimental setup of DESI and DEFFI MSI platforms. (**A**) Schematic diagram of DESI sprayer. (**B**) Detailed information of the DESI sprayer head outlined in panel (**A**). (**C**) Schematic diagram of DEFFI sprayer. (**D**) Detailed information of the DEFFI sprayer head in panel (**C**).

MSI experiments were carried out with a self-made DESI and DEFFI source, which have been reported before [8]. The DESI probe was lab-built with a 1/16″ stainless-steel T-junction (Figure 1A,B). The nebulizer tip consisted of two concentric fused silica capillaries: an outer 350/250 μm (O.D./I.D.) sheath gas capillary and an inner solvent capillary of 150/20 μm (O.D./I.D.). Capillaries were hand-cut with a ceramic stone, and great care was taken to ensure an even, blunt end. The sheath gas and solvent capillaries were both secured in the T-junction using graphite or graphite/Vespel ferrules. The solvent capillary extended continuously from the nebulizer tip, through the T-junction, and ended in a high-pressure PEEK union assembly with Fingertight ferrules (1/16″ tubing O.D. with 0.010″ I.D. thru hole, 10–32). The solvent capillary was secured into one end of the PEEK union with a NanoTight sleeve (1/16″ O.D. × 0.007″ I.D.). A short length of PFA tubing (1/16″ O.D. × 0.030″ I.D.) was secured in the other end of the union and served as the inlet for the solvent syringe. During the imaging process, the Y-distance between the DESI tip and the sample surface was set as 4 mm. The X-distance between the DESI tip and the MS inlet was set as 2 mm. The impact angle between the DESI sprayer and the sample stage was set at 60°. Compressed N_2_ (120 psi, 99.999% purity) was used as the nebulizing gas. Mass spectra were recorded in both negative-ion mode and positive-ion mode. The injection rate of the DESI solvent (95% methanol in water) was set at a different flow rate as indicated before. An Orbitrap mass spectrometer (Fusion, Thermo Fisher Scientific, Waltham, MA, USA) was used to obtain MS data. Positive and negative full MS scans were employed over the range of *m*/*z* 150–1000. The automatic gain control (AGC) was set to the off position to keep the scan rate constant. The MS inlet capillary temperature was set at 300 °C. MS images were generated using a self-coded program running in MATLAB (2021b, Mathworks, Natick, MA, USA).

For DEFFI, experiments were performed using a flow-focusing sprayer design on which the solvent emitter is retracted inside a grounded spray head, so that the electrospray is formed and electro-hydrodynamically focused within the sprayer before exiting as a narrow droplet jet. This implementation follows the general DEFFI concept reported for Orbitrap-based MSI, where grounding the spray orifice and applying high voltage to the solvent establish an internal electric field that stabilizes and focuses the spray. As schematized in Figure 1C,D, the DEFFI sprayer consisted of a solution capillary (fused silica, 350/100 μm O.D./I.D.) housed in a stainless-steel tube and terminated in front of a conductive spray head held at electrical ground. The spray head contained a small circular orifice that served as both the flow-focusing nozzle and the grounded electrode. N_2_ (30 psi) was supplied to the sprayer body and directed coaxially around the emitter toward the nozzle, providing pneumatic assistance and hydrodynamic focusing of the emerging spray. High voltage (1500 V for both positive and negative mode) was applied to the solvent upstream, while the spray head remained grounded, producing a well-defined electric field inside the nozzle region. The focused spray plume was directed toward the sample surface and aligned to the mass spectrometer ion transfer tube for efficient ion capture. Operational parameters (solvent flow rate, nitrogen pressure, spray voltage, sprayer–sample distance, and sprayer–inlet distance) were optimized for each experiment to maximize signal stability while maintaining the desired spatial resolution.

## 3. Results

### 3.1. Experimental Setup of DESI and DEFFI MSI Platforms

Figure 1 shows the experimental configurations used to perform DESI-MSI and DEFFI-MSI on the same ambient MSI platform. In both cases, a pneumatically assisted spray of charged solvent droplets was directed onto the sample surface to induce desorption and ion formation, and the generated ions were subsequently sampled by the mass spectrometer through the ion transfer tube positioned adjacent to the sampling region.

For conventional DESI-MSI, the sprayer employed a coaxial capillary design consisting of an inner solvent capillary and an outer sheath-gas capillary [8]. High voltage was applied to the solvent to produce charged primary droplets, while high-purity nitrogen (99.999%) served as the nebulizing gas to assist droplet formation and accelerate the spray toward the sample (Figure 1A). The enlarged view in Figure 1B shows the detailed information of the DESI sprayer head: the inner fused silica capillary (150/20 μm, O.D./I.D.) was concentrically positioned within an outer capillary (350/250 μm, O.D./I.D.), sealed with a stainless-steel ferrule and a polymer sleeve. The resulting droplet plume attached on the surface to generate secondary droplets that carried desorbed analyte ions toward the MS inlet.

For DEFFI-MSI, the sprayer geometry which has been reported before was used in this study (Figure 1C) [21]. The solvent was delivered through a solution capillary (350/100 μm, O.D./I.D.) held inside a stainless-steel tube and inserted into a grounded spray head (Figure 1D). Nitrogen was introduced into the spray head and flowed coaxially around the capillary to pneumatically focus the emerging liquid at the emitter tip. With the solvent maintained at high voltage relative to the grounded spray head, the combined electric field and gas focusing produced a narrow, high-velocity stream of charged microdroplets directed toward the sample. Similar to DESI, analyte-containing secondary droplets generated at the surface were sampled through the ion transfer tube for mass spectrometric detection.

### 3.2. Comparison of Spatial Resolution of DESI and DEFFI MSI

A 7 µm thick mouse brain cryosection was selected as a sample to benchmark the lateral resolving capability of DESI-MSI and DEFFI-MSI. Rather than equating spatial resolution with the programmed pixel size, we quantified the effective resolution using an edge-response definition that is widely adopted in laser-based MSI [22,23,24,25]. This distinction is important for droplet-based ambient imaging because the pixel size only specifies the sampling grid, whereas the measurable resolution is ultimately limited by the physical interaction spot on the surface (wetting/spreading and secondary droplet formation), which can remain substantially larger than the pixel pitch.

**Figure 2:**
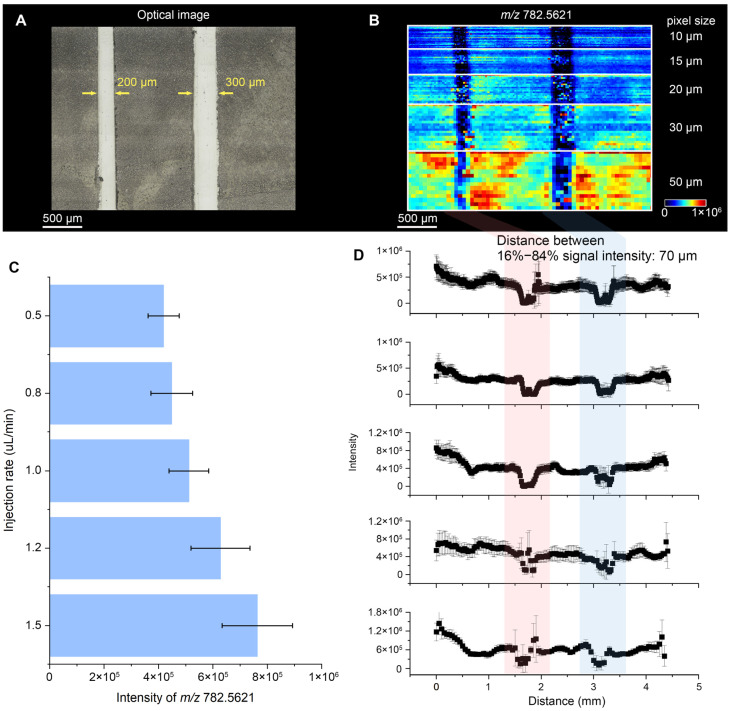
Evaluation of spatial resolution of DESI. (**A**) Optical image of the DESI-MSI area, in which two gaps were made. (**B**) MS image of the ion at *m*/*z* 782.5621 with a pixel size of 10, 15, 20, 30, and 50 μm. (**C**) Intensity of the ion at *m*/*z* 782.5621 at different solution injection rates. (**D**) Line profiles across both gaps of the ion at *m*/*z* 782.5621.

Spatial resolution was defined as the distance over which the ion signal changes from 16% to 84% of its maximum when the spray scans a sharp boundary, which is a widely adopted and robust way to quantify lateral resolution in MSI. In this method, the spatial resolution is inferred from the spread edge function rather than from nominal pixel size. The 16–84% criterion measures the distance over which the signal rises across a nominally sharp boundary while avoiding the low-intensity tails (e.g., <16%), which are more susceptible to baseline drift and noise, and the near-plateau region (>84%), which can be affected by local heterogeneity and saturation. In both DESI and DEFFI configurations, the solvent flow rate primarily controls the droplet flux and the amount of solvent delivered to the surface, thereby affecting surface spreading and the effective sampling spot. Lower flow rates tend to reduce the interaction footprint and improve spatial localization, but they also decrease analyte extraction and ion yield, leading to weaker signals. Conversely, higher flow rates increase extraction efficiency and signal intensity but can broaden the effective footprint due to increased wetting and splash dynamics, which degrades the effective spatial resolution. The solvent flow rate was varied from 0.5 to 1.5 µL min^−1^ (0.5, 0.8, 1.0, 1.2, and 1.5 µL min^−1^), and the stage velocity was adjusted accordingly to produce nominal pixel sizes of 10, 15, 20, 30, and 50 µm. Two well-defined gaps (200 and 300 µm in width) were introduced on the tissue section (Figure 2A), and the region was imaged by DESI in positive-ion mode. The resulting ion images of *m*/*z* 782.5621 (Figure 2B) show that decreasing the pixel size improves visual sharpness, consistent with denser spatial sampling, yet this comes with an evident loss in signal abundance. The intensity trend is summarized in Figure 2C, where the *m*/*z* 782.5621 signal decreases as the solvent flow rate is lowered. To extract an objective resolution value, line profiles across both gaps were generated from the ion images (Figure 2D). For each condition, five adjacent scan lines were combined (*n* = 5) to reduce random variability and provide a robust estimate of the edge transition width. Notably, although the pixel size was reduced to 10 µm, the narrowest 16–84% transition obtained with DESI was 70 µm, indicating that the effective lateral resolution of this DESI configuration is capped at ~70 µm under the tested conditions. Increasing sampling overlap by further slowing the stage can densify coverage, but it does not compress the 16–84% transition beyond this limit, consistent with a resolution ceiling imposed by the intrinsic DESI sampling spot rather than by the programmed pixel size.

The solvent flow rate window (0.5–1.5 µL/min) was selected because it represents the practical operating range that balances spray stability, ion yield, and effective spatial resolution for our setup. At higher flow rates, we observed increased surface wetting and a larger effective spray–surface interaction spot, which is expected to broaden the edge transition and therefore degrade the effective spatial resolution. So, we did not pursue flow rates above 1.5 µL/min for the resolution benchmarking. At lower flow rates, solvent delivery from the syringe pump became unstable in our configuration, leading to fluctuating ion signals and poor reproducibility; therefore, flow rates below 0.5 µL/min were not systematically evaluated. In principle, using a higher-precision delivery system (e.g., a syringe pump optimized for sub-µL/min operation, or a nano liquid chromatography pump with improved flow stability) could enable stable operation at lower flow rates and may further reduce the effective sampling footprint, potentially yielding additional gains in spatial resolution.

**Figure 3:**
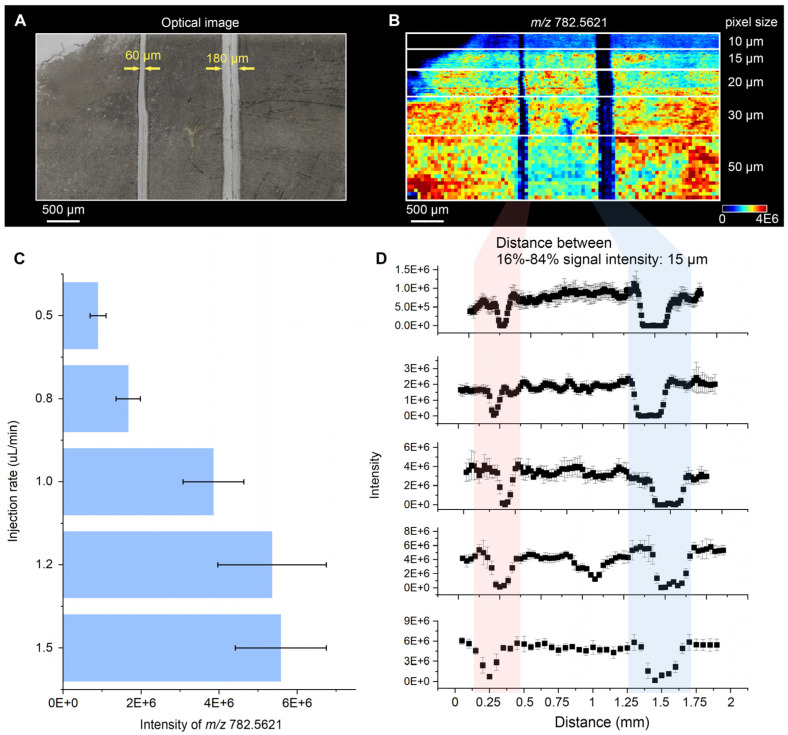
Evaluation of spatial resolution of DEFFI. (**A**) Optical image of the DESI-MSI area, in which two gaps were made. (**B**) MS image of the ion at *m*/*z* 782.5621 with a pixel size of 10, 15, 20, 30, and 50 μm. (**C**) Intensity of the ion at *m*/*z* 782.5621 at different solution injection rates. (**D**) Line profiles across both gaps of the ion at *m*/*z* 782.5621.

Using the same 7 µm mouse brain cryosection format and the same edge-response metric, DEFFI-MSI was evaluated under an analogous set of operating conditions to determine whether a reduced effective sampling spot could be achieved without sacrificing ion yield. The solvent flow rate was again varied from 0.5 to 1.5 µL min^−1^ (0.5, 0.8, 1.0, 1.2, and 1.5 µL min^−1^), and the stage velocity was adjusted to generate nominal pixel sizes of 10, 15, 20, 30, and 50 µm. For DEFFI, two narrower gaps were patterned on the tissue section to challenge the resolving capability (nominal widths of 60 and 180 µm; Figure 3A), and the region was imaged in positive-ion mode using *m*/*z* 782.5621 as the representative signal.

Across the full set of pixel sizes, DEFFI produced ion images with visibly sharper boundaries than those obtained with DESI (Figure 3B), and the gap features remained well-defined even when the nominal pixel size approached the scale of the narrower gap. Unlike the DESI case, signal loss at reduced flow was less limiting for imaging contrast: the intensity of *m*/*z* 782.5621 remained high over the tested flow-rate window (Figure 3C), indicating a larger usable operating window where spatial sampling can be densified without collapsing signal. The quantitative resolution was extracted from line profiles across both gaps (Figure 3D), calculated from five adjacent scan lines (*n* = 5) to suppress stochastic pixel-to-pixel variation. The resulting 16–84% transition distance reached 15 µm, demonstrating that DEFFI reduces the effective interaction spot to a scale close to the smallest programmed pixel sizes used here. In this regime, decreasing pixel size improves image sharpness in a manner that more directly reflects true resolution, consistent with a sampling spot-limited ceiling that is substantially lower than that observed for DESI under comparable imaging constraints.

Overall, applying the same 16–84% edge-response definition on identical 7 µm mouse brain cryosections reveals a clear difference between the two droplet-based MSI configurations. Conventional DESI exhibits a sampling spot-limited resolution ceiling of ~70 µm under the tested conditions, such that reducing the pixel size primarily increases sampling density without further narrowing the edge transition. In contrast, DEFFI achieves a substantially smaller effective interaction region and reaches a 15 µm lateral resolution (~4.7× improvement), while maintaining a broader operating window in which high image contrast can be preserved. These results establish that DEFFI provides a more favorable resolution–signal trade-off for high-resolution ambient MSI.

### 3.3. MSI of Maize Root Sections

**Figure 4:**
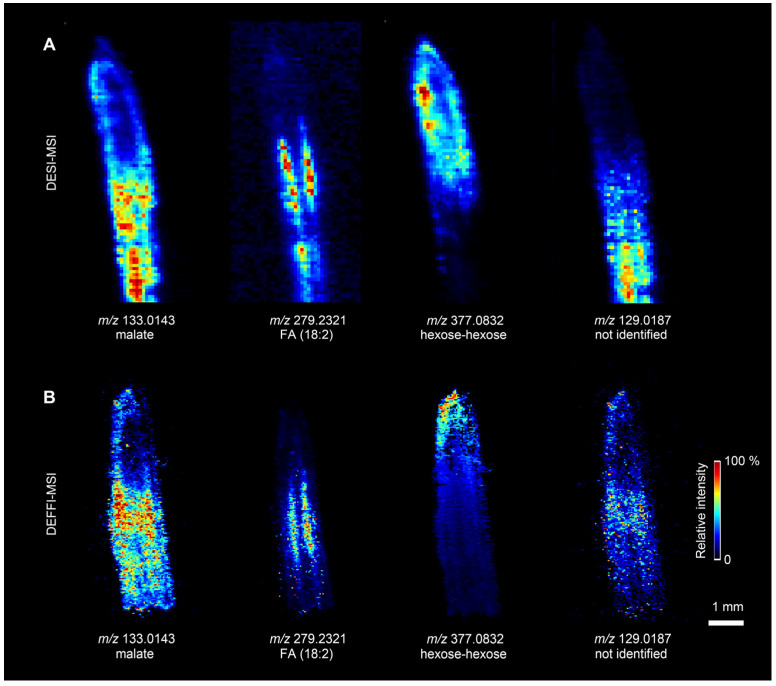
Comparison of DESI-MSI and DEFFI-MSI on a maize root section. (**A**) Representive ion images of *m*/*z* 133.0143, *m*/*z* 133.279.2321, *m*/*z* 377.0832, and *m*/*z* 129.0187 by DESI-MSI. (**B**) MS images of the same ions by DEFFI-MSI.

To evaluate practical imaging performance on a challenging, small biological specimen, we compared DESI-MSI and DEFFI-MSI on a thin maize root section with a sub-millimeter-scale cross section (Figure 4). Maize root has well-defined yet narrow anatomical layers, making it a sensitive test for effective spatial resolution and chemical contrast [8]. In DESI-MSI (Figure 4A), ion distributions of several representative features (*m*/*z* 133.0143, 279.2321, 377.0832, and 129.0187) were detectable but appeared broadened and less confined to specific tissue compartments, which limited the ability to resolve fine structures across the root radius. In contrast, DEFFI-MSI (Figure 4B) produced markedly sharper and more compartmentalized ion patterns, enabling clearer separation of major root tissues, including the stele, quiescent center, endodermis, cortex, epidermis, and root cap. The improved structural delineation is consistent with the reduced effective sampling footprint and higher ion yield characterized above, and it highlights the benefit of DEFFI for high-resolution ambient MSI when the biological morphology is defined by narrow layers. Notably, compared with prior DESI-based maize root MSI studies, the DEFFI images here provide higher apparent anatomical contrast on this thin and geometrically confined sample, strengthening the case for DEFFI as a practical upgrade for resolving tissue microstructures in plant MSI.

### 3.4. Comparison of Maximum Ion Injection Time of DESI and DEFFI

**Figure 5:**
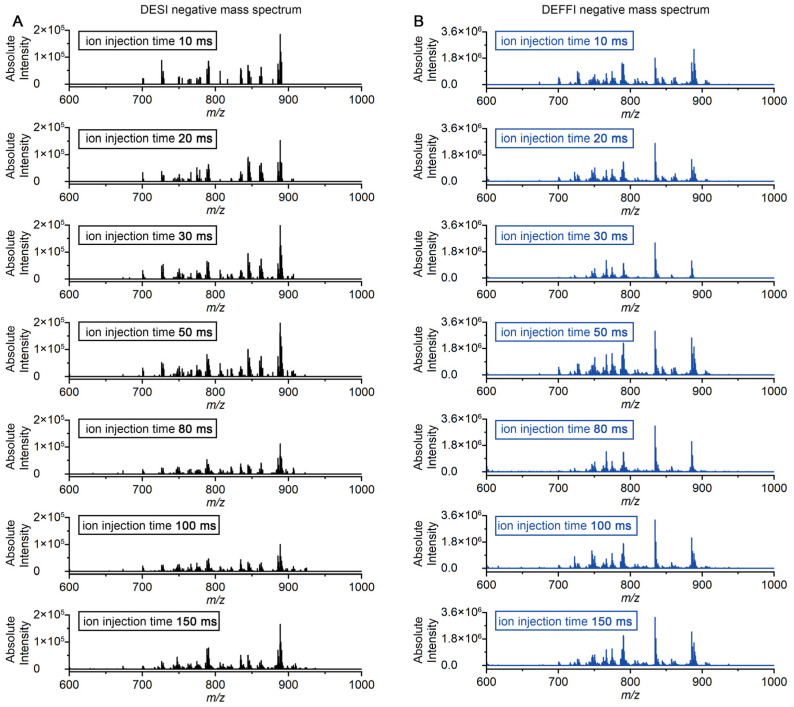
Comparison of the MS signal intensity under negative-ion mode by (**A**) DESI and (**B**) DEFFI with different ion injection times ranging from 10 ms to 150 ms.

**Figure 6:**
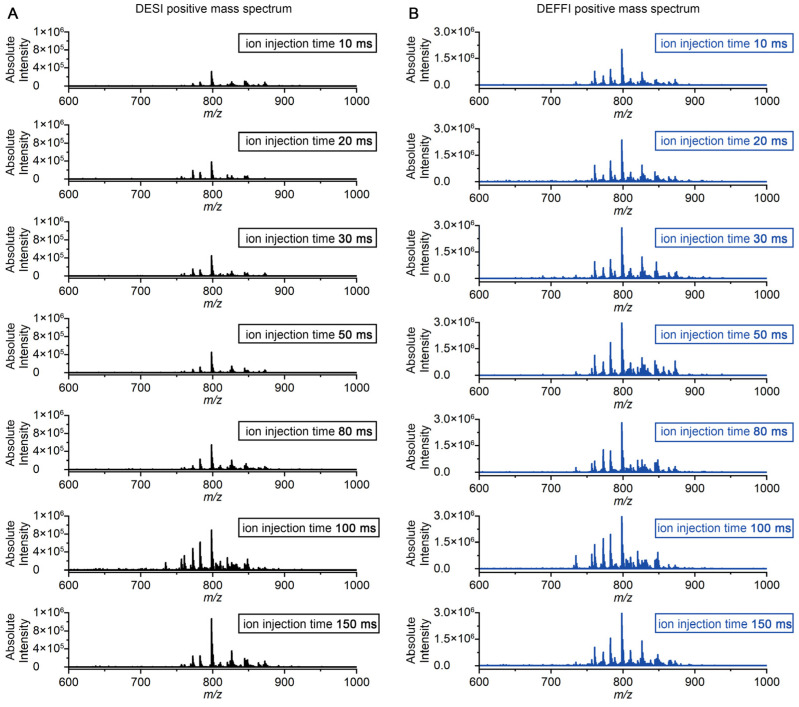
Comparison of the MS signal intensity under positive-ion mode by (**A**) DESI and (**B**) DEFFI with different ion injection time from 10 ms to 150 ms.

Orbitrap-based MSI is frequently limited by ion accumulation because the mass spectrometer must collect sufficient ions before each transient. To directly evaluate how ion yields from DESI and DEFFI translate into Orbitrap collection cycles, we compared the full-scan spectral output while systematically decreasing the maximum ion injection time from 150 ms to 10 ms. The experiment was performed in both negative-ion mode (Figure 5) and positive-ion mode (Figure 6) to test whether the trend was polarity-dependent. The data were collected on the same mouse brain tissue sample used before.

As shown in Figure 5A, under negative-ion mode, DESI produced relatively sparse spectra at short injection times, indicating that sufficient ion collection cannot be achieved within a short period of time. Although the highest peak intensity is consistent with the long-collection-time mode, the spectrum exhibits significant loss in low-abundance ion signals. Increasing the maximum injection time increased the number of detectable ions and their intensities, indicating that additional accumulation time is required to build a usable signal for DESI-MSI. In contrast, DEFFI generated rich spectra with substantially higher signal abundance even when the maximum injection time was set to 10 ms (Figure 5B). The improvement was maintained across the entire injection-time range tested, demonstrating that DEFFI delivers a much higher ion flux to the Orbitrap inlet under the same constraints. In Orbitrap-based MSI, the achievable imaging throughput is typically constrained by the mass spectrometer duty cycle rather than by the translation stage speed. Under our acquisition conditions, the scan rate is dominated by ion accumulation: at a resolving power setting of 30,000, a maximum ion injection time of 150 ms yields an effective acquisition frequency of ~2.6 Hz, whereas reducing the maximum injection time to 10 ms increases the acquisition frequency to ~10 Hz with the same resolving power. Because DEFFI maintains strong spectral output at short injection times, it allows routine operation in this high-frequency regime, translating into an approximately fourfold increase in practical imaging speed compared with DESI.

Similar behavior was also observed in positive-ion mode (Figure 6). When the maximum injection time was reduced, DESI spectra rapidly lost intensity and spectral richness at 10–30 ms (Figure 6A), whereas DEFFI retained strong spectral output and preserved abundant peaks at the same short injection times (Figure 6B). The consistent advantage in both polarities indicates that the benefit of DEFFI is not restricted to a specific ion class but instead reflects a general increase in ion production. The higher ion yield observed for DEFFI is consistent with several previous studies [20,21]. In the DEFFI geometry, the solvent capillary is retracted behind the orifice and combined with gas-assisted flow focusing, which produces a spray that is more tightly focused and rotationally symmetric than conventional DESI at comparable flow rates. Flow focusing can generate a steady liquid jet that breaks into micron-sized, relatively monodisperse droplets downstream, and applying a voltage between the feed capillary and a grounded orifice plate (“electro-flow focusing”) can further reduce droplet size while keeping the potential drop largely self-contained near the nozzle. This configuration may reduce radial plume divergence (including space-charge-driven expansion) and increase the fraction of charged droplets efficiently delivered toward the surface and subsequently into the MS inlet, leading to higher effective ion flux.

Overall, these results show that DEFFI can operate effectively at very short maximum ion injection times (10 ms) while maintaining high spectral quality, whereas DESI requires substantially longer injection times to approach comparable detectability. Because injection time directly contributes to the Orbitrap duty cycle per pixel, the higher ion flux provided by DEFFI enables markedly faster MSI acquisition without sacrificing spectral content, consistent with the throughput improvements expected for high-resolution Orbitrap imaging.

## 4. Discussion

This study shows that a previously reported DEFFI-MSI configuration provides clear practical advantages over conventional DESI-MSI on an Orbitrap platform, most prominently in effective spatial resolution, signal intensity, ion yield, and imaging throughput. Although both approaches rely on pneumatically assisted charged droplets to desorb and ionize analytes at atmospheric pressure, their performance diverges when evaluated using objective resolution metrics and under injection-time-limited acquisition conditions.

A key finding is that nominal pixel size does not necessarily represent the true resolving capability of droplet-based MSI. Using the MALDI-MSI-style 16–84% edge-response definition on 7 µm mouse brain cryosections, DESI reached a spot-limited resolution ceiling of ~70 µm: decreasing the pixel size increased the sampling density but did not further compress the edge transition. In contrast, DEFFI achieved a 15 µm edge response (~4.7× improvement), indicating a substantially smaller effective surface interaction spot. DEFFI also generated higher signal intensity across the tested solvent flow-rate range, reducing the typical resolution–signal trade-off that often restricts high-resolution DESI operation.

The higher ion yield from DEFFI translates directly into faster Orbitrap imaging. Orbitrap throughput is frequently limited by ion accumulation rather than stage motion, because insufficient ion flux forces longer injection times to reach the desired ion population. In both negative- and positive-ion modes, DEFFI maintained rich, full-scan spectra even when the maximum injection time was reduced to 10 ms, whereas DESI spectra weakened markedly under the same constraint. Under our conditions at a resolving power of 30,000, shortening the maximum injection time from 150 ms to 10 ms increased the acquisition frequency from ~2.6 Hz to ~10 Hz, enabling an approximately fourfold gain in practical imaging speed when DEFFI is used.

DEFFI’s performance gains can be rationalized by considering how spray physics and droplet-mediated desorption ionization jointly determine the surface interaction footprint and the effective ion flux delivered to the MS inlet. The electro-flow-focusing geometry used in DEFFI (a retracted emitter behind a grounded orifice with gas-assisted focusing) is expected to generate a more collimated, rotationally symmetric droplet jet with reduced radial divergence, thereby shrinking the effective footprint and producing sharper edge responses. At the same time, the higher signal intensity and the ability to operate at very short injection times suggest that DEFFI increases the fraction of analyte-containing charged droplets/ions that are transferred into the inlet, which may arise from improved spray focusing and a more localized potential drop near the nozzle that reduces plume expansion. Because Orbitrap throughput is directly controlled by the ion flux available during accumulation, this enhanced delivery efficiency provides a consistent physical explanation for the observed improvements in both spatial resolution and injection-time-limited imaging speed.

## 5. Conclusions

The improvements of DEFFI shown in this study broaden the application space of ambient MSI on high-resolution instruments. A higher effective resolution supports clearer delineation of fine tissue microstructures, as illustrated on the thin maize root sample, where DEFFI better distinguishes narrow anatomical layers. Higher ion yield improves robustness and sensitivity, benefiting low-abundance species and reducing the need for aggressive parameter tuning. Faster acquisition makes high-mass-resolution imaging more feasible for larger areas and higher replicate numbers, strengthening the utility of MSI for comparative biological and translational studies. The higher spatial resolution and faster acquisition make DEFFI competitive in broad applications, for example, in single-cell spatial metabolomics, environmental analysis, and imaging analysis related to large clinical samples.

## Data Availability

Data is available on reasonable request from the corresponding authors.
